# “We’ll check vital signs only till we finish the school”: experiences of student nurses regarding intra-semester clinical placement in Ghana

**DOI:** 10.1186/s12912-018-0292-0

**Published:** 2018-05-29

**Authors:** Charles Ampong Adjei, Collins Sarpong, Priscilla Adumoah Attafuah, Ninon P. Amertil, Yaw Abayie Akosah

**Affiliations:** 1 Department of Community Health Nursing, School of Nursing, College of health Sciences, University of Ghana, Accra, Ghana; 2 Department of Nursing, Valley View University, Box AF 595, Adenta, Accra, Ghana

**Keywords:** Experience, Baccalaureate, Student nurses, Intra-semester, Clinical practicum

## Abstract

**Background:**

Clinical practicum is an integral part of nursing education because it provides students with opportunities to perform nursing care and practice specific nursing tasks. In Ghana, little is known about the experiences of baccalaureate student nurses with regard to intra-semester clinical practicum. This study therefore, explored perceptions, challenges, and how the intra-semester clinical practicum affects the learning process of student nurses in a private university in Ghana.

**Methods:**

Exploratory descriptive phenomenological design was used. Nine in-depth interviews and three focus group discussions were conducted for baccalaureate student nurses in their second, third and fourth years of study. Only those who have attended intra-semester clinical practicum for at least two semesters in the course of their study were recruited. Purposive sampling technique was used to select the participants. The sample size was based on data saturation, however, a total of 33 participants were recruited. Data was analysed using content analysis technique.

**Results:**

The findings show that baccalaureate student nurses perceive the intra-semester clinical practicum as beneficial. It affords the opportunity to translate theoretical knowledge into practice concurrently. However, students recounted their stressful experiences during the clinical period which negatively affected their academic work. Additionally, staff nurses assigned the students to do menial jobs instead of appropriate nursing tasks.

**Conclusions:**

A review of the “block” method in which students will go to clinicals for a stipulated number of consecutive days in a month and then resume lectures, is worth considering.

**Electronic supplementary material:**

The online version of this article (10.1186/s12912-018-0292-0) contains supplementary material, which is available to authorized users.

## Background

Nursing is a practice-based profession and therefore the clinical setting is an essential part of the training [1–3].The preparation of student nurses involves rigorous theoretical and practical instruction [4]. However, effective training is dependent on appropriate supervision and mentorship particularly in the clinical environment [5–7].

It is well established that student nurses perceive clinical placement as rewarding [8] since it has the tendency to improve their clinical skills, connect theory with practice and support their professional growth [8–12]. Notwithstanding, the clinical environment presents numerous challenges to students [3, 13–15]. For instance, in South Africa, nursing students complained of lack of support by staff nurses [15]. Some incidents of bullying of student nurses were documented in other countries [14, 16]. These incidents created anxiety and depression among the students and consequently affected the care they offered to their clients. Despite all these occurrences, the overall positive effect of clinical placement of nursing students cannot be underestimated.

Furthermore, different nursing practical methodologies are used by various nursing schools all over the world. For example, in the United Kingdom (UK), student nurses are placed in the clinical learning environment for 6 weeks continuously which enables them in gaining self-confidence and improves their communication with clients and nursing staff [17]. What is unique about this approach is that registered nurses who participate in the Nursing and Midwifery Council’s (NMC) approved mentorship programme, support the students professionally [17]. On the contrary, South African student nurses are in the clinical environment for only 14 days, a time period which they perceive as too short for acquisition of nursing skills [18].

In Ghana, a recent curriculum designed by the NMC requires each student nurse to obtain clinical contact of 432 h, 624 h, and 576 h during the first, second and third year of training respectively [19].This curriculum allows nursing schools in the country to adopt either of the acceptable clinical placement schedules; intra-semester and inter-semester clinical placement [19].

For the past 3 years, Valley View University has implemented these two methodologies prescribed by the council. During vacation, students are assigned to hospitals for clinical attachment for a maximum of 8 weeks (inter-semester practicum). Then when classes are in session, the students attend lectures 3 days in a week and spend the remaining 2 days in the clinical setting to ensure that they satisfy the NMC’s intra-semester practicum requirement. This means that teaching and practical attachment are done concurrently during the course of the semester. This study therefore was aimed at documenting the perception and challenges that students face during intra-semester clinical placement since the limited literature in Ghana focuses primarily on student’s attitude toward clinical work [2].

## Methods

### Study design

This study used exploratory descriptive phenomenological design. Considering the fact that intra-semester clinical practicum is a new practical methodology used by the University, this design was best suited to explore the experiences of the students.

### Study setting

The study was conducted in the Greater Accra region of Ghana. The region is the capital town of the country and shares borders with the Eastern region to the north, Central region to the west, Volta region to the east and the Gulf of Guinea to the south [20]. About 4,010,050 people reside in the region [20].The study was conducted at Valley View University which was established in 1979 by the West Africa Union Mission of the Seventh-Day Adventist Church. The baccalaureate nursing programme of the institution commenced in September, 2007. Currently, it has a student population of 462.

### Participant’s eligibility

#### Inclusion criteria

Participants were included in the study if they were baccalaureate student nurses who have completed at least two semesters of their 4 year programme and have attended intra-semester clinical practicum at least twice in the course of their study and consented to participate.

#### Exclusion criteria

First year baccalaureate student nurses were excluded.

### Sample and sampling methods

Participants were selected purposively. In all, 33 participants took part in the study. The study objectives were shared with the students in class. Those who were interested to participate and met the inclusion criteria were recruited. Nine in-depth interviews made up of three students each from year two, three and four were conducted. In addition, three focus group discussions, which consisted of eight students from each of the three levels, were done.

### Data collection tool

A semi-structured interview guide was used to conduct the in-depth interviews and the focus group discussions. The guide had both open and closed ended questions such as: “How do you perceive intra-semester clinical practicum?”; “Did the clinical practicum have any effect (positive or negative) on your learning?”; “Did you face any challenges with intra-semester clinical practicum?” The guide was designed based on literature and experts’ contributions (refer to Additional file 1).

### Data procedure

Data collection started between June and August, 2016 following ethical clearance and permission from University authorities. On the day of the scheduled interview/discussion, the researchers met the participants at the agreed venues which included classrooms and conference rooms in the school. The study aims were explained to the participants and their written consents were obtained. In addition, their consent to audio record the interviews/discussions were sought. The in-depth interviews lasted between 45 min and 1 h. However, the focus group discussions lasted between 60 min and 90 min. None of the participants experienced any psychological disturbances in the course of narrating their experience.

### Data analysis

Data were analysed using content analysis technique. The recorded interviews were played and listened to by the researchers. Each researcher coded the data, which was followed by a series of group discussions by the research team. Finally, major themes and sub-themes were generated and presented as findings of the study.

### Methodological rigour

Rigour refers to trustworthiness. Rigour in qualitative study must satisfy the following criteria: credibility, transferability, dependability, and confirmability [21]. In this study, credibility was achieved by piloting the interview guide using baccalaureate student nurses who shared similar characteristics but were not part of the study participants. Transferability was ensured by the use of direct quotes from participants and a thick description of the study setting. An audit trail with the voice records, transcripts, field notes and diaries was kept to ensure dependability and confirmability.

### Ethic approval

The study was ethically approved by the Dodowa Health Research Center of the Ghana Health Service (protocol Number-DHRCIRB/07/06/16). Permission was also sought from the Head of the Nursing Department of the University. In addition, participants’ written consents were obtained after the rationale of the study was explained. Pseudonyms are used to ensure anonymity of the participants.

## Results

### Socio-demographic characteristics

The study involved thirty three (33) baccalaureate student nurses in their second, third and final year of the nursing programme. They ranged in age from 19 to 24 years. Each participant had attended intra-semester clinical practicum at least twice in the course of their study. In all, three themes and seven sub-themes emerged from the data and are presented below.

#### Perception about intra-semester clinical practicum

This theme describes how the students perceived intra-semester clinical practicum. A number of the participants indicated that the practicum was very beneficial. However, some added that it was associated with stress, which consequently affected their academic work.

### Benefits of intra-semester clinical practicum

The study revealed how clinical practicum presents an opportunity for student nurses to appreciate what they are taught in the classroom, particularly courses that are practically oriented. Some shared their thoughts:

*“Intra-semester clinical is very helpful because aside the lectures and the skills lab, you need a real patient to try your hands on. It is only on the ward that you get such cases and time to practice.”* **[Kofi − 300]**

*“Intra-semester clinical is important. For example, you can only appreciate a course like community health nursing when you see it being practiced and not that you learn and wait till a certain period of time before you go and practice.”* **[Akua − 300]**

Notwithstanding, some students indicated that they only derived the full benefit of the clinical practicum when they were assigned to wards that offered care to patients with conditions that were in line with the topics being discussed in class.

*“When you are placed on a ward that is related to what you are studying in class, it helps you better understand the theoretical aspects of the nursing course”* **[Afia − 400]**

### Stress

Almost all the participants during the interview expressed their concerns about the stressful nature of the intra-semester clinical practicum. According to the participants, long distance to the hospitals and the fact that they must attend lecture the next day compounds the situation.

They lamented:

*“Going for the clinical is stressful, I mean very stressful. The long distance from the school to the hospital makes it very stressful.”* **[Adwoa-300]**

*“Going for afternoon shift especially is very tiring. We come back from clinical very late in the evening meanwhile, we have lectures early in the morning on the next day. So I think it’s too stressful.”* **[Akosua-400]**

### Effects on academic performance

Some participants echoed the effect of the intra-semester clinical on their academic performance. According to them, they were unable to prepare adequately for class the next day especially when they returned to campus very late. The carry over effect of the stress also influenced most students to sleep in class during lecture periods.

*“...when you go for afternoon shift and come around 9pm, you will be tired, and learning your books before the next lectures becomes very difficult. You are unable to revise your lecture notes and so you get confused when you’re taught new things in class”* **[Kwaku-400]**

*“We mostly sleep in class because of the tiredness we experience. Also, we don’t have time to read on our previous lessons”* **[Akua − 300]**

#### Learning environment

The study revealed that there is a huge gap between what students learn in class and what is being practiced in the hospitals. In addition, some participants reported that they were not supported by the staff nurses.

### Theory and practice gap

The majority of the participants indicated that a gap existed between theory and practice. According to them, the nurses in the hospitals did not follow standard protocols in providing nursing care. This contradicts the systematic way in which they are taught in the school’s skill laboratory. Some shared their frustrations:

*“The procedures that they use at the wards mostly don’t follow protocol like how we’re taught in the school’s skills laboratory. The way they* (nurses) *do things on the ward is different. They* (the nurses) *want to be able to serve everybody quickly and therefore do not follow the exact protocol that we’ve been taught”* **[Nii-200]**

One participant argued why it may be better to learn the practical component of nursing from the literature rather than going to the hospital. He said:

*“Sometimes I think it seems better reading the literature and getting things right about practical aspect of nursing than going to the ward where improvisation is the theme of the day. You can imagine if you pick the wrong things there by doing them, it’ll be very difficult to conform to standards”* **[Kojo-400]**

### Student-nurse relationship

Some participants mentioned that the learning environment was not friendly due to a lack of bonding between the students and the staff nurses. This was attributed to the fact that students were placed in the hospital twice a week and therefore were unable to acquaint themselves very well with the staff nurses.

*“…because the practicum is not continuous, there is absence of that kind of close relationship and cordial relationship with the nurses.”* **[Akweley-300]**

Furthermore, some participants expressed that the poor relationship had a negative impact on their skill acquisition on the ward. A student said:

*“Because we go twice a week, we’re always new on the ward so the ward in-charges do not have confidence in us to let us do much of the procedures. They think we still don’t know…, we’re still novice on the ward because they don’t see us every day.”* **[Kwabena − 400]**

One participant was afraid that continuing with just the 2 day contacts in a week at the hospital will not give him the chance to practice advanced nursing procedures but only the checking of vital signs.

*“When we continue to go twice a week, we’ll always check vital signs only till we finish the school.”* **[Kofi-200]**

#### Challenges of intra-semester clinical practicum

This theme describes the challenges that confronted the students during the intra-semester clinical practicum. Some participants indicated that they were only used for menial jobs in the hospitals. Others also mentioned that the twice a week contact at the hospital did not afford them opportunity to know the outcome of their patients’ conditions.

### Break in continuity of care

The study revealed that most participants were unable to follow their patient’s progress to the end and therefore were not able to measure their success.

*“When we go twice a week and we care for patients, it takes another week before we meet them again. Sometimes, it is very difficult to follow up… you have to pick a new patient again.”* **[Kwesi-300]**

*“when you’re on the ward and you nurse a patient for a particular week, by the time you go for the following week you’ll realize that the patient has been discharged and you can’t get access to the folder so it seems you’ve done nothing for the patient.”* **[Adwoa-400]**

### Messenger role

A number of the participants expressed how the staff nurses used them for menial jobs on the ward instead of allowing them to spend productive hours with their patients.

Yaw had this to say:

*“They* (nurses) *kept on sending me everywhere, I went to the orthopedic unit, and many places. Anytime I return they will say that I have the long legs so I have to be transporting patients to other units. Sometimes, I don’t even get time with the patients. I sometimes spent a maximum of five minutes with my patients the whole day.”* **[Yaw-200]**

## Discussion

This study explored the experiences of baccalaureate student nurses regarding intra-semester clinical practicum. The findings showed that students perceived intra-semester clinical practicum as beneficial since it afforded the opportunity to translate theory into practice concurrently as previously reported in other studies [8, 10, 22]. For instance, in Saudi Arabia, about 75.6% (*n* = 205) of nursing students were satisfied with their clinical practicum [10]. The similarities may have resulted from the fact that student nurses recognised nursing as both an art and science and therefore understood the importance of the practical component.

The study also found that clinical placement was associated with stress. The long distance (35.5 km) to the hospitals and early morning class schedules for the next day, compounded the participants’ stressful experiences. Previous studies have also documented stress as one challenge that negatively affected the learning processes and the general health of nursing students [12, 23, 24].According to Jamshidi et al. [23],student nurses in Iran were stressed by the new experience of being in the hospital and also by the complexities of devices they observed on patients. Other related studies supported the experiences of stress in the clinical environment for students [24, 25].It is worth considering repackaging the clinical placement schedule such that students spend sometime in the classsroom and subsequently halt for practicum during the course of the semester.

Many studies have reported on the existence of a gap between theory and practice in relation to nursing education [8, 18]. Some participants in this study mentioned that the systematic way of carrying out nursing procedures was missing in the hospitals. Students were therefore in a state of dilemma as to whether or not they should copy the practices of staff nurses, which were not evidence supported. The non-compliance to standard procedures by the staff nurses is not surprising since it appears there is an imbalance in the nurse-to-patient ratio which increases the workload of nurses. Also, we can speculate that some nurses are not confident enough to demonstrate nursing procedures to students. Clinical supervisors need to be guided by clinical objectives before assigning students to the hospital wards. In addition, preceptors must be trained and assigned to students for supervision and assessment. This will bridge the extensively reported gap between theory and practice.

The study also found that the learning environment was unsupportive and this was evidenced by the poor relationship that existed between students and staff nurses. This corroborated a study by Mabuda, Potgieter& Alberts [15] which found that nurses failed to support students during clinical attachment. Perhaps the behaviour of students, including lateness to work, the use of mobile phones on duty, lack of commitment to clinical work and absenteeism without permission as documented in a similar study in Ghana [2], might have influenced the reaction of the staff nurses toward the students. It is therefore important to orient students on their clinical objectives before commencing their clinical schedules. It is also important to have the students followed by experienced clinical instructors to ensure the best learning results [2]. Faculty support in the clinical environment has significant impact, particurlarly in facilitating, evaluating and monitoring the students during the clinical period [13], and therefore it is worth strengthening. Moreover, staff nurses need to recognise themselves as mentors and important stakeholders in the training of student nurses.

The findings further showed that some staff nurses treated the students as messengers instead of learners. It was widely reported that student nurses were used for errands and were further tasked to do menial jobs instead of providing nursing care. This finding is not peculiar to Ghana but is also present in other countries [3, 18]. This obviously has an implication for nursing skill acquisition since the students are not encouraged to take on challenging tasks to develop their professional skills. It is important to assign students to specific tasks as soon as they report to the hospital and these tasks should be evaluated at the end of the shift by a preceptor or clinical instructor.

## Conclusions

The findings of this study provide insight to nursing students’ experiences in the clinical environment. It reveals that student nurses perceive intra-semester clinical practicum as a good approach for acquisition of skills. However, there are some challenges that require an immediate review of the methodology so as to help students derive the full benefit of the intra-semester clinical practicum. One suggestion may be an adoption of the “block” method where students will go for clinical attachment for a stipulated number of consecutive days in a month and then resume lectures.

## Additional file

Additional file 1: Interview guide (DOCX 14 kb)

## Abbreviation

NMC: Nursing and Midwifery Council of Ghana
