# Supplementary material for: “We’ll check vital signs only till we finish the school”: experiences of student nurses regarding intra-semester clinical placement in Ghana
Source: BMC Nurs. 2018 May 29;17:23. doi: 10.1186/s12912-018-0292-0 (PMC5975683; doi:10.1186/s12912-018-0292-0)
Supplement: Supplementary file 1 — Interview guide (DOCX 14 kb) [file 12912_2018_292_MOESM1_ESM.docx]

**INTERVIEW GUIDE**

**Demographic Data**

Please can you tell me about yourself?

Age:

Sex:

Level:

How Many Years of Clinical Placement:

**Perception**

1. Please can you share with me what you think about clinical placement as part of your nursing program? (Probe)

**Learning process**

1. Can you share with me how the intra-semester clinical placement is affecting your learning positively? (Probe)
2. Can you tell me how the intra-semester clinical placement is affecting your learning negatively? (probe)

**Challenges**

1. Please can you share with me the challenges that you are faced with during the clinical placement? (probe)
